# Risk of cardiovascular disease following gonadotropin‐releasing hormone agonists vs antagonists in prostate cancer: Real‐world evidence from five databases

**DOI:** 10.1002/ijc.33397

**Published:** 2020-11-23

**Authors:** Gincy George, Hans Garmo, Lucie‐Marie Scailteux, Frédéric Balusson, Greet De Coster, Harlinde De Schutter, Josephina G. Kuiper, Emmanuel Oger, Julie Verbeeck, Mieke Van Hemelrijck

**Affiliations:** ^1^ King's College London, Translational Oncology and Urology Research London UK; ^2^ University of Rennes, EA 7449 REPERES Pharmacoepidemiology and Health Services Research Rennes France; ^3^ Rennes Hospital University, Pharmacovigilance Pharmacoepidemiology and Drug Information Center Rennes France; ^4^ Belgian Cancer Registry Brussels Belgium; ^5^ PHARMO Institute for Drug Outcomes Research Utrecht The Netherlands

**Keywords:** cardiovascular disease, GnRH agonists, GnRH antagonists, prostate cancer, real‐world evidence

## Abstract

Observational studies in prostate cancer (PCa) have shown an increased risk of cardiovascular disease (CVD) following gonadotropin‐releasing hormone (GnRH) agonists, whereas randomised‐controlled trials have shown no associations. Compared to GnRH agonists, GnRH antagonists have shown less atherosclerotic effects in preclinical models. We used real‐world data from five countries to investigate CVD risk following GnRH agonists and antagonists in PCa men. Data sources included cancer registries, primary and secondary healthcare databases. CVD event was defined as an incident or fatal CVD. Multivariable Cox proportional hazard models estimated hazard ratios (HRs) and 95% confidence intervals (CIs), which were pooled using random‐effects meta‐analysis. Stratified analyses were conducted by history of CVD and age (75 years). A total of 48 757 men were on GnRH agonists and 2144 on GnRH antagonists. There was no difference in risk of any CVD for men on GnRH antagonists and agonists (HR: 1.25; 95% CI: 0.96‐1.61; *I* ^2^: 64%). Men on GnRH antagonists showed increased risk of acute myocardial infarction (HR: 1.62; 95% CI: 1.11‐2.35; *I* ^2^: 0%) and arrhythmia (HR: 1.55; 95% CI: 1.11‐2.15, *I* ^2^: 17%) compared to GnRH agonists. Having a history of CVD was found to be an effect modifier for the associations with some CVD subtypes. Overall, we did not observe a difference in risk of overall CVD when comparing GnRH antagonists with agonists—though for some subtypes of CVD we noted an increased risk with antagonists. Further studies are required to address potential confounding caused by unadjusted variables such as severity of CVD history and PCa stage.

## Abstract 2

**What's new?**

Prolonged use of gonadotropin‐releasing hormone (GnRH) agonists has been associated with an increased risk of cardiovascular disease (CVD). Preclinical studies have indicated that newer GnRH antagonists may cause fewer atherosclerotic effects than GnRH agonists. In this meta‐analysis of prostate cancer studies, however, the authors found little benefit of GnRH antagonists over GnRH agonists, in terms of CVD risk. If anything, men taking GnRH antagonists may have an increased risk of acute myocardial infarction and arrhythmia compared to GnRH agonists. Further studies are warranted.

**Abbreviations**
ADT: androgen deprivation therapy
AMI: acute myocardial infarction
BCR: Belgian Cancer Registry
BMI: body mass index
CI: confidence interval
CVD: cardiovascular disease
FDA: Food and Drug Administration
FSH: follicle‐stimulating hormone
GnRH: gonadotropin‐releasing Hormone
HF: heart failure
HR: hazard ratio
ICD: International Classification of Diseases
IHD: ischaemic heart disease
NHSS: National Health Service Scotland
PCa: prostate cancer
PMSI: National Hospital‐discharge Summaries database system
PRONOUNCE: A Trial Comparing Cardiovascular Safety of Degarelix vs Leuprolide in Patients with Advanced Prostate Cancer and Cardiovascular Disease
PSA: prostate‐specific antigen
RCT: randomised‐controlled trial
ROBINS‐I: Risk of Bias in Non‐Randomised Studies of Interventions
SAS: statistical analysis software
SES: socio‐economic status
SNIIRAM: Système National d'Informations Inter‐Régimes de l'Assurance Maladie
STATA: statistics and data
THIN: The Health Improvement Network

## 1 INTRODUCTION

In 2010, the United States Food and Drug Administration (FDA) issued a requisite for gonadotropin‐releasing hormone (GnRH) agonists, a main form of androgen deprivation therapy (ADT) for locally advanced and metastatic prostate cancer (PCa), to carry a safety warning on the drug labels after several observational studies,^1^, ^2^, ^3^, ^4^, ^5^, ^6^, ^7^ and a meta‐analysis of observational studies^8^ showed an increased risk of cardiovascular disease (CVD) in individuals on GnRH agonists. Degarelix, a GnRH antagonist introduced in 2010, has been associated with a lower risk of CVD in men with PCa.^9^, ^10^ Preclinical models have shown less atherosclerotic effects in mice treated with the GnRH antagonist as compared to those treated with GnRH agonists.^11^ Although GnRH agonists are a GnRH inhibitor, GnRH antagonists are a GnRH blocker that completely blocks GnRH receptors. The difference in mechanism of action leads to an immediate mode of action in GnRH antagonists associated with its reduced side effects.^12^

Phase II and phase III studies showed no difference in terms of efficacy and baseline testosterone levels in men receiving GnRH antagonists for 1 year compared to men receiving various GnRH agonists for their PCa.^13^ Comparison of the CVD safety profile in men on GnRH agonists and antagonists has yielded inconclusive results.^8^, ^14^ Although meta‐analysis of observational studies^8^ have shown a lower risk of CVD in men on GnRH antagonists compared to GnRH agonists, meta‐analyses of randomised‐controlled trials (RCTs) have shown no such associations.^14^ Moreover, these studies were not designed with CVD as a primary outcome.

A phase III RCT (PRONOUNCE; ClinicalTrials.gov identifier: NCT02663908) is recruiting to compare the risk of fatal or nonfatal CVD in 900 men with PCa receiving GnRH agonist or antagonist as primary treatment.^15^ Results of the PRONOUCE trial is not expected until 2021, which justifies the need for observational evidence in the interim. Since RCTs often exclude elderly participants and those with comorbidities, two most common characteristics of men with PCa receiving ADT,^16^ real‐world data used in observational studies may provide evidence applicable to the general PCa population.^17^, ^18^ Only one observational study has been conducted to date directly comparing risk of CVD between GnRH agonists and GnRH antagonists. Scailteux et al showed no difference in risk of developing stroke or myocardial infarction in men with PCa receiving either treatments; however, overall CVD was not investigated as a specific outcome in the study.^19^

By combining real‐world data from five countries, we designed a study with sufficient power to compare risk of CVD between GnRH agonists and antagonists in a real‐world setting. Our study is the first observational study to directly compare the risk of six CVD outcomes between GnRH agonists and antagonists by using country‐specific analyses from patient level data from five countries. We explored six CVD outcomes: any CVD, ischaemic heart disease (IHD), acute myocardial infarction (AMI), arrhythmia, heart failure (HF) and stroke.

## 2 METHODS

### 2.1 Study design and population

This observational study combined data from five countries to investigate the association between GnRH agonists or antagonist (degarelix) and risk of CVD in men with PCa. All men with PCa prescribed or dispensed GnRH agonists or GnRH antagonists were included in the study. A detailed study protocol aimed to minimise heterogeneity in terms of data collection across countries by outlining exact codes for extraction of all study variables is published elsewhere.^20^

Data from the United Kingdom's The Health Improvement Network (THIN) database (excluding Scotland)^21^, ^22^ (2010‐2016), National Health Service Scotland (NHSS)^23^ (2010‐2017), the Belgian Cancer Registry (BCR)^24^ (2010‐2015), the PHARMO Database Network from the Netherlands^25^ (2010‐2015) and the French Système National d'Informations Inter‐Régimes de l'Assurance Maladie (SNIIRAM)^26^ (2010‐2013) were used for our study. Although the THIN database is a primary healthcare database (ie, general practices and community healthcare settings) and NHSS is a secondary healthcare database (ie, hospitals and outpatient clinics), BCR and PHARMO include both primary and secondary healthcare data.^21^, ^22^, ^23^, ^24^, ^25^, ^26^, ^27^ The SNIIRAM database is a claims database combining claims from insurance plans with the National Hospital‐discharge Summaries database system (PMSI).^26^ As Scotland is in the United Kingdom and there may have been some overlap of men with PCa in the THIN database and Scottish NHSS database, men with PCa with a postcode in Scotland were excluded from THIN. The study period extended from 2010 to 2017 in the five countries.

### 2.2 Exposures

Exposure was defined as a prescription or dispensation of GnRH agonists or GnRH antagonists. We included all men with locally advanced or metastatic PCa (in countries where PCa stage was available, Supplement Table 1) who started on GnRH agonists or GnRH antagonists. Men were followed from the date of GnRH agonists or antagonist's initiation until outcome of interest, switch between agonists and antagonists and vice versa, orchiectomy, end‐of‐study period or death from other causes, whichever came first. No further exclusion criteria were used.

### 2.3 Outcome

A CVD event was classified as first (incident or fatal) CVD. Six specific CVD outcomes were explored: any CVD (International Classification of Diseases [ICD]‐10: I20‐I99, G45), IHD (ICD‐10: I20‐I25), AMI (ICD‐10: I21), arrhythmia (ICD‐10: I44‐I49), HF (ICD‐10: I50, I97.710, I97.790, I11.0) and stroke (ICD‐10: I60‐64, G45). In Belgium, ICD‐9 equivalent codes were used in cases where no ICD‐10 classification was available. In the United Kingdom, the THIN database made use of already published readcodes instead of ICD codes (used in NHSS, BCR, PHARMO and SNIIRAM).^28^

### 2.4 Other study variables

In addition to age, we obtained information on the following covariates to better understand data heterogeneity across countries: follow‐up time, year of PCa diagnosis, stage of PCa, total Gleason score, prostate‐specific antigen (PSA), any prior PCa treatment, type of ADT, ADT specifics, history of CVD indicator and number of previous CVD events (Table 1 and Supplement Table 1). Additional information on socio‐demographic variables, such as socio‐economic status (SES), civil status and ethnicity and lifestyle factors, including body mass index (BMI) and smoking, were available in THIN. History of CVD indicator was classified as any CVD event or prescription or dispensation of medication for hypertension, dyslipidaemia or diabetes within 12 months prior to GnRH initiation. Only CVD events or risk factors for CVD in the past 12 months were captured. Detailed description and codes for other study variables have been described elsewhere.^20^

**TABLE 1:** Baseline characteristics for men with prostate cancer from the five included databases in the United Kingdom (excluding Scotland), Scotland, Belgium, the Netherlands and France

Demographic or clinical characteristic	United Kingdom (excluding Scotland)		Scotland		Belgium		Netherlands		France	
	Men on GnRH agonists	Men on GnRH antagonists	Men on GnRH agonists	Men on GnRH antagonists	Men on GnRH agonists	Men on GnRH antagonists	Men on GnRH agonists	Men on GnRH antagonists	Men on GnRH agonists	Men on GnRH antagonists
	N (%)	N (%)	N (%)	N (%)	N (%)	N (%)	N (%)	N (%)	N (%)	N (%)
Study period	2010 to 2016		2010 to 2017		2010 to 2015		2010 to 2015		2010 to 2013	
Number of men with PCa	16 955 (99.3)	118 (0.7)	9114 (94.8)	495 (5.2)	1860 (78.1)	522 (21.9)	1187 (92.5)	97 (7.6)	19 641 (83.9)	912 (3.9)
Follow‐up time, years										
Median	0.6	0.5	2.1	0.8	1.7	1.1	2.3	1.5	2.2	2.1
Lower quartile	0.2	0.1	1.1	0.6	0.8	0.5	1.5	1.2	0.7	0.8
Upper quartile	1.8	1.2	2.9	1.1	3.0	1.8	3.4	2.2	2.7	2.6
Age, years										
Mean	75	74	73	74	73.5	72.3	71.9	72.5	74.2	73.4
SD	9.6	10.1	8.4	9.2	9.3	9.8	8.3	9.6	8.6	9.8
≤65	1627 (9.6)	21 (17.8)	1641 (18.0)	84 (10.9)	390 (21.0)	130 (24.9)	276 (23.3)	24 (24.7)	3016 (15.4)	191 (20.9)
66 to 74	3543 (20.9)	43 (36.4)	3895 (43.0)	192 (25.0)	555 (29.8)	162 (31.0)	452 (38.1)	33 (34.0)	6358 (32.4)	278 (30.5)
75 to 84	4322 (25.5)	33 (28.0)	1852 (20.3)	99 (12.9)	697 (37.5)	177 (33.9)	387 (32.6)	26 (26.8)	8124 (41.4)	318 (34.9)
≥85	1901 (11.2)	21 (17.8)	1726 (18.9)	120 (15.6)	218 (11.7)	53 (10.2)	72 (6.1)	14 (14.4)	2143 (10.9)	125 (13.7)
Missing	5562 (32.8)	0	0	0	0	0				
History of CVD indicator										
Yes	8288 (48.9)	70 (59.3)	2876 (31.6)	119 (24.0)	1364 (73.3)	361 (69.2)	741 (62.4)	64 (66.0)	14 011 (71.3)	625 (68.5)
No	8667 (51.1)	48 (40.7)	6238 (68.4)	376 (76.0)	496 (26.7)	161 (30.8)	446 (37.6)	33 (34.0)	5630 (28.7)	287 (31.5)

Abbreviations: CVD, cardiovascular disease; GnRH, gonadotropin‐releasing hormone; PCa, prostate cancer.

### 2.5 Statistical analysis

We conducted a two‐stage analysis: firstly, we obtained country‐specific hazard ratios (HRs) from Cox proportional hazards models using age as a timescale to assess heterogeneity in each country, and secondly, we conducted a meta‐analysis. The Cox models included stratified analysis (history of CVD indicator and age at cut‐off point of 75 years) from each country with age as a timescale. The country‐specific HRs from Stage 1 were then combined in a random‐effects meta‐analytic model assessing the risk of any CVD and CVD subtypes comparing men on GnRH agonists with men on GnRH antagonists. The *I* ^2^ statistic was calculated to determine the proportion of variation in the estimates due to heterogeneity. To assess the effect of heterogeneity, we then performed a sensitivity analysis excluding the United Kingdom as the data were collected using readcodes—as compared to ICD codes in Scotland, Belgium, the Netherlands and France. We also conducted stratified analyses by history of CVD indicator and age (<75 and ≥75 years). An age of 75 years was used as a cut‐off point in the study because the mean age was 73 or 74 years. Moreover, men above 75 years may be more likely to present with very advanced disease and this difference was accounted for by the analysis.^29^

A further sensitivity analysis was also conducted to exclude men who may have been on GnRH agonists or GnRH antagonists for less than 3 months to eliminate short‐term neoadjuvant or adjuvant use.

All country‐specific analyses were conducted using different SAS versions in Belgium (9.4) and France (9.4) and statistics and data (STATA) versions in the United Kingdom (14C), Scotland (14) and the Netherlands (14C). The meta‐analysis was conducted using STATA version 14C.

## 3 RESULTS

Table 1 shows the baseline and clinical characteristics of men on GnRH agonists or GnRH antagonists in the United Kingdom, Scotland, Belgium, the Netherlands and France. A total of 48 757 men were on GnRH agonists from the United Kingdom (n = 16 955), Scotland (n = 9114), Belgium (n = 1860), the Netherlands (n = 1187) and France (n = 19 641). Of which, 2144 men were on GnRH antagonists from the United Kingdom (n = 118), Scotland (n = 495), Belgium (n = 522), the Netherlands (n = 97) and France (n = 912). Total median follow‐up time for the United Kingdom, Scotland, Belgium, the Netherlands and France was 1.8 (0.9‐2.8) years for GnRH agonists and 1.2 (0.6‐1.8) years for GnRH antagonists. Total mean age for the five countries was 74 (SD = 8.8) for GnRH agonists and 73 (SD = 9.7) for GnRH antagonists.

Socio‐demographic factors were only available in the United Kingdom and high missing numbers for BMI, SES, smoking status and civil status resulted in an exclusion of these variables from the analytical models. Moreover, clinical characteristics of PCa were also not uniformly available in all countries. We reported these variables for descriptive purposes and no further analyses were conducted using these variables.

**FIGURE 1:**
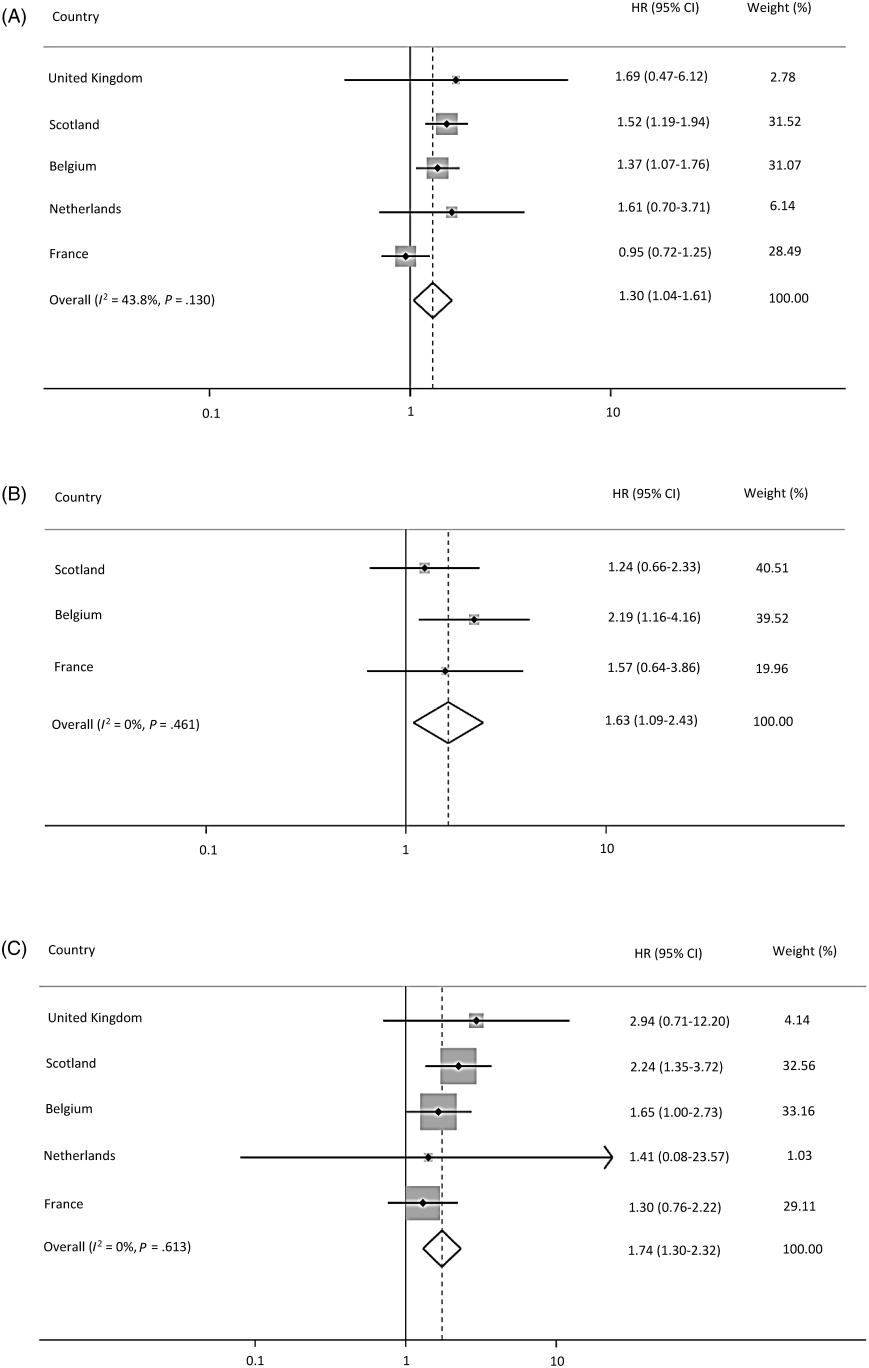
A, Pooled results from meta‐analysis for prostate cancer (PCa) men with a history of cardiovascular disease (CVD) indicator developing any CVD including United Kingdom, Scotland, Belgium, the Netherlands and France. B, Pooled results from meta‐analysis for PCa men with a history of CVD indicator developing acute myocardial infarction including Scotland, Belgium and France. C, Pooled results from meta‐analysis for PCa men with a history of CVD indicator developing arrhythmia including United Kingdom, Scotland, Belgium, the Netherlands and France

Table 2 shows pooled HRs including stratifications to evaluate the use of GnRH agonists compared to GnRH antagonists according to history of CVD indicator and age. HRs shown were derived from a random‐effects meta‐analysis model including the United Kingdom, Scotland, Belgium, the Netherlands and France. With respect to our primary objective, there was no increased risk of developing any CVD in both comparison groups (HR = 1.25; 95% CI = 0.96‐1.61; *I* ^2^ = 64%). However, men with PCa on GnRH antagonists had an increased risk of developing AMI (HR = 1.62; 95% CI = 1.11‐2.35; *I* ^2^ = 0%) and arrhythmia (HR = 1.55; 95% CI = 1.11‐2.15; *I* ^2^ = 17%) compared to men on GnRH agonists. An increased risk was observed for any CVD (HR = 1.30; 95% CI = 1.04‐1.61; *I* ^2^ = 44%; Figure 1A), AMI (HR = 1.63; 95% CI = 1.09‐2.43; *I* ^2^ = 0%; Figure 1B) and arrhythmia (HR = 1.74; 95% CI = 1.30‐2.32; *I* ^2^ = 0%; Figure 1C) for men on GnRH antagonists with a history of CVD. For men who were on GnRH antagonists without a history of CVD indicator, there was an increased risk of developing arrhythmia (HR = 5.37; 95%; CI = 1.26‐22.87; *I* ^2^ = 0%) compared to those on GnRH agonists.

**TABLE 2:** Hazard ratios from random‐effects meta‐analytical models including different stratification for any CVD, ischaemic heart disease, acute myocardial infarction, arrhythmia, heart failure and stroke for the five included countries

Outcome	HR (95% CI)	HR for PCa men with^a^ history of CVD indicator (95% CI)	HR for PCa men without history of CVD indicator (95% CI)	HR for PCa men < 75 years (95% CI)	HR for PCa men ≥ 75 years (95% CI)
Any CVD	1.25 (0.96–1.61)	1.30 (1.04–1.61)	1.15 (0.77‐1.73)	1.29 (1.00‐1.65)	1.16 (0.92‐1.46)
Ischaemic heart disease	1.22 (0.95‐1.58)	1.18^b^ (0.82‐1.69)	1.85 (1.00‐3.41)	1.17^c^ (0.81‐1.69)	1.31^b^ (0.94‐1.82)
Acute myocardial infarction	1.62^d^ (1.11‐2.35)	1.63^d^ (1.09‐2.43)	2.05^e^ (0.75‐5.62)	2.16^d^ (1.27–3.67)	1.31^d^ (0.77‐2.22)
Arrhythmia	1.55 (1.11‐2.15)	1.74 (1.30‐2.32)	5.37^f^ (1.26‐22.87)	1.62 (1.04–2.51)	1.44^b^ (1.00‐2.08)
Heart failure	1.34 (0.97‐1.85)	1.33^b^ (0.94‐1.87)	2.45^g^ (0.85‐7.05)	1.71^d^ (0.91‐3.21)	1.22 (0.83‐1.80)
Stroke	0.88 (0.60‐1.29)	0.86^d^ (0.54‐1.36)	1.44^b^ (0.69‐3.01)	0.79^d^ (0.30‐2.06)	0.94 (0.60‐1.47)

*Note*: GnRH agonists is the reference group in all analyses.

Abbreviations: CI, confidence interval; CVD, cardiovascular disease; GnRH, gonadotropin‐releasing hormone; HR, hazard ratio; PCa, prostate cancer.

^a^ History of CVD indicator was defined as a prescription or dispensation of medication for any of the following 12 months prior to entering the cohort: any CVD event, hypertension, dyslipidaemia or diabetes.

^b^ The Netherlands was excluded due to low number of events for country‐specific analysis.

^c^ The United Kingdom was excluded due to low number of events for country‐specific analysis.

^d^ The United Kingdom and the Netherlands were excluded due to low number of events for country‐specific analysis.

^e^ The United Kingdom, Belgium and the Netherlands were excluded due to low number of events for country‐specific analysis.

^f^ Scotland, the Netherlands and France were excluded due to low number of events for country‐specific analysis.

^g^ The United Kingdom, Scotland and the Netherlands were excluded due to low number of events for country‐specific analysis.

For men aged <75 years and on GnRH antagonists, there was an increased risk of developing AMI (HR = 2.16; 95% CI = 1.27‐3.67; *I* ^2^ = 0%) and arrhythmia (HR = 1.62; 95% CI = 1.04‐2.51; *I* ^2^ = 0%). No remarkable differences in results were observed when excluding the United Kingdom (Supplement Table 2) compared to the main analysis (Table 2). Sensitivity analysis with GnRH initiation date 3 months after the GnRH start date showed no statistically significant findings for arrhythmia (Supplement Table 3).

## 4 DISCUSSION

Our study is the first to combine real‐world data from the United Kingdom, Scotland, Belgium, the Netherlands and France to compare the risk of CVD following GnRH agonists and GnRH antagonists in men with PCa. Our results from all five countries provided no support for a difference in risk of any CVD between GnRH antagonists compared to GnRH agonists, but there was some evidence of increased risk of certain CVD subtypes. Men with PCa given GnRH antagonists with history of CVD event or indication had a 30% higher chance of developing any CVD, 63% higher chance of developing AMI and 74% higher chance of developing arrhythmia compared to men on GnRH agonists. Stratification by age showed that men on GnRH antagonists aged <75 years had 62% higher chance of developing arrhythmia compared to men on GnRH agonists.

Our study showed that there was an increased risk of developing any CVD, AMI and arrhythmia in men on GnRH antagonists with a prior CVD history compared to GnRH agonists. Our final results are in contrast to our preliminary findings^30^ using four of the five countries, which looked at the proportion of men developing a CVD event in the exposure groups, without accounting for age or follow‐up period. The difference in methods may have reflected the results as we pooled country‐specific HRs in our final meta‐analysis of five countries.

This is the first study to show an increased risk of developing CVD subtypes in men on GnRH antagonists compared to GnRH agonists. Although this is significant, it is important to note that degarelix, the GnRH antagonist, was a new drug during the study period with strict prescription guidelines that tailored the drug to specific PCa population (ie, to those with preexisting CVD). Even though we have accounted for history of CVD by history of CVD indicator stratified analysis, we may have selected a population with underlying CVD risk leading to an increased risk of CVD in men on GnRH antagonists observed in our study. Moreover, we only accounted for history of CVD indicator 12 months prior to GnRH initiation due to data unavailability, which may also have limited the number of CVD events in both arms of the study. The generic history of CVD indicator variable may have led to a loss of granularity in terms of assessment of history of CVD. This may have contributed to confounding by indication in our study due to the inability to adjust for precise history and severity of prevalent CVD. Moreover, men with more advanced stage PCa who are given more aggressive forms of PCa treatments may already have had elevated CVD risks due to their disease stage and treatments. By not accounting for PCa stage in our study, we may have missed some of the information indicative of treatment history and potential increased CVD risks.

Our findings showing an increased risk of CVD subtypes in men on GnRH antagonists compared to GnRH agonists contradicts prior literature showing an increased CVD risk among men using the GnRH agonists compared to GnRH antagonists.^9^, ^10^, ^11^, ^31^ Although GnRH agonists work by inhibiting the release of GnRH, antagonists work by inhibiting both GnRH and follicle‐stimulating hormone (FSH) receptors.^32^, ^33^, ^34^ The additional inhibition of FSH receptors by GnRH antagonists may reduce the risk of a recurrent CVD due to FSH receptors' role in lipid metabolism and in fat accumulation.^35^ However, results from our real‐world data showed contradictory effects, which may be partly explained due to confounding by indication.

In our methodological protocol, we used the Risk of Bias in Non‐Randomised Studies of Interventions (ROBINS‐I) tool to assess our study design, which emphasised three main forms of biases: misclassification of study variables, channelling or indication and unmeasured confounding.^20^ We avoided misclassification bias, where possible, by following a standard protocol^20^ to extract study variables from the five countries involved. Channelling or indication bias^36^ occurs when a physician prescribes GnRH antagonists to men with a history of CVD based on previous evidence.^37^ We were unable to avoid channelling bias in our study because data on physician preferences were not available. In addition to no information on physician preference, heterogeneity in guidelines for prescription of GnRH antagonists in the five countries may have also affected the study results (discussed in George et al^20^).

In this large prospective cohort study, we found it difficult to fully homogenise study variables (Supplement Table 4). A large proportion of this complexity was attributed to the varied data sources used in our study. THIN from the United Kingdom was the most distinct database used in the study, due to its data derivation exclusively from primary healthcare settings.^21^ As degarelix was a fairly new drug on the market^38^ and a much smaller proportion of men were degarelix users in individual countries, there was a need to combine data from all countries in our analysis. We attempted to account for this heterogeneity in the data sources by not only setting up a standard protocol^20^ but also by focusing analyses on data that was fully available in all five countries (ie, history of CVD indicator). Moreover, further sensitivity analyses excluding the United Kingdom (Supplement Table 2) and a delayed start date of 3 months after GnRH initiation date (Supplement Table 3) showed little difference in patterns for risk of various CVD types.

Heterogeneity in data sources at the point of the data capture process may also reflect the results of our study. Neither healthcare records nor claims databases have research as a primary intention at point of data capture. Although claims databases reflect diagnosis codes recorded to justify medical prescriptions and procedures, electronic healthcare records reflect data captured to support clinical care that may not represent a complete medical history.^39^

Results for some subtypes of CVD were limited due to the data sources that they were obtained from. For example, the acute nature of AMI means that it is usually recorded in an acute hospital setting.^40^ The United Kingdom had no AMI events for analysis because the THIN database originates from a primary healthcare setting. Therefore, further assessment of hospital registries is needed to understand the risk of developing AMI in men with PCa on GnRH analogues.

A key strength of our study was the use of data from five countries that made study results applicable to the general PCa population. Moreover, the use of different types of databases (primary healthcare, secondary healthcare, cancer registries and claims databases) also ensured the inclusion of rare, adverse events that may not have been identified in an RCT. However, our study also highlighted the challenges involved of using real‐world data. Although the potential for real‐world data is large in the healthcare setting, differences in data sources need supervised reconfiguration of data for real‐world data to achieve its full potential. The way forward for researchers using real‐world data is to combine and analyse “big data” from databases from various institutions into a single platform through projects such as the GetReal Initiative^41^ and Prostate cancer dIagnOsis and treatmeNt Enhancement through the power of big data in EuRope (PIONEER),^42^ which are part of the Big Data for Better Outcomes (BD4BO).^43^

## 5 CONCLUSION

Our study across five countries provided little support for a difference in risk of any CVD between GnRH antagonists compared to GnRH agonists, but there was some evidence of increased risk of certain CVD subtypes. Since our results are based on real‐world data, they may be more applicable to the general PCa population who are on hormonal treatment. However, the potential for indication bias in our study needs to be addressed through RCTs, such as the PRONOUNCE trial.

## CONFLICT OF INTEREST

JGK is an employee of the PHARMO Institute for Drug Outcomes Research, which is an independent research institute that performs financially supported studies for government and related healthcare authorities and pharmaceutical companies. The other authors declare that there is no conflict of interest.

## DISCLAIMER

The views expressed in this article are the author(s) own and not that of any particular institution or funder.

## ETHICS STATEMENT

Ethical approval was obtained for all data used in the study from relevant ethical committees and healthcare authorities in the five countries.

## Supporting information


**Appendix** **S1**: Supporting Information

## Data Availability

The data are not publicly available due to privacy or ethical restrictions. The results presented in this article are from the meta‐analysis (stage two) which combine country‐specific hazard ratios as combining data from all countries at an individual level was not possible due to ethical and legal restrictions on data sharing. Datasets that are minimally required to replicate the outcomes of the study will be made available upon reasonable request.
