# Supplementary material for: Risk of cardiovascular disease following gonadotropin‐releasing hormone agonists vs antagonists in prostate cancer: Real‐world evidence from five databases
Source: Int J Cancer. 2020 Nov 23;148(9):2203–11. doi: 10.1002/ijc.33397 (PMC8049028; doi:10.1002/ijc.33397)
Supplement: Supplementary file 1 — Appendix S1: Supporting Information [file IJC-148-2203-s001.pdf]

Full title: Risk of cardiovascular disease following GnRH agonists versus antagonists in prostate cancer: Real-world evidence from five databases.

Authors: Gincy George, Hans Garmo, Lucie-Marie Scailteux, Frédéric Balusson, Greet De Coster, Harlinde De Schutter, Josephina G. Kuiper, Emmanuel Oger, Julie Verbeeck, Mieke Van Hemelrijck

Contents

Supplement Table 1: Baseline characteristics for men with prostate cancer from the five included databases in the United Kingdom (excluding Scotland), Scotland, Belgium, the Netherlands and France	………………………………	Pages 2-11
Supplement Table 2: Hazard ratios from sensitivity analysis excluding UK using random-effects meta-analytical model including different stratifications any CVD, ischaemic heart disease, acute myocardial infarction, arrhythmia, heart failure and stroke for Scotland, Belgium, the Netherlands and France.	………………………………	Page 12
Supplement Table 3: Hazard ratios from sensitivity analysis for 3 months using random-effects meta-analytical model including different stratifications any CVD, ischaemic heart disease, acute myocardial infarction, arrhythmia, heart failure and stroke for the United Kingdom, Scotland, Belgium, the Netherlands and France.	………………………………	Page 13
Supplement Table 4: Data extraction in the United Kingdom, Scotland, Belgium, the Netherlands and France.	………………………………	Pages 14-15

Supplement Table 1: Baseline characteristics for men with prostate cancer from the five included databases in the United Kingdom (excluding Scotland), Scotland, Belgium, the Netherlands and France.

Demographic or Clinical Characteristic	United Kingdom (excluding Scotland)		Scotland		Belgium		Netherlands		France	
	Men on GnRH Agonists	Men on GnRH Antagonists	Men on GnRH Agonists	Men on GnRH Antagonists	Men on GnRH Agonists	Men on GnRH Antagonists	Men on GnRH Agonists	Men on GnRH Antagonists	Men on GnRH Agonists	Men on GnRH Antagonists
	N (%)	N (%)	N (%)	N (%)	N (%)	N (%)	N (%)	N (%)	N (%)	N (%)
**Year of PCa diagnosis**										
< 2010	1815 (10.7)	4 (3.4)	0	0			0	0	N/A	N/A
2010	1719 (10.1)	4 (3.4)	1490 (16.3)	8 (1.6)	496 (26.7)	41 (7.9)	256 (21.6)	27 (27.8)	N/A	N/A
2011	1508 (8.9)	14 (11.9)	1638 (18.0)	23 (4.6)	494 (26.6)	108 (20.7)	275 (23.1)	23 (23.7)	N/A	N/A
2012	1582 (9.3)	13 (11.0)	1431 (15.7)	60 (12.1)	433 (23.3)	143 (27.4)	236 (19.9)	14 (14.4)	N/A	N/A
2013	1532 (9.0)	15 (12.7)	1614 (17.7)	91 (18.4)	437 (23.5)	230 (44.1)	238 (20.1)	22 (22.7)	N/A	N/A
2014	1241 (7.3)	24 (20.3)	1535 (16.8)	166 (33.5)	0	0	182 (15.3)	11 (11.3)	N/A	N/A

2015	821 (4.8)	31 (26.3)	1406 (15.4)	147 (29.7)	0	0	0	0	N/A	N/A
2016	183 (1.1)	7 (5.9)	0	0	0	0	0	0	N/A	N/A
Missing	6,554 (38.7)	6 (5.1)	0	0	0	0	0	0	N/A	N/A
**Stage of PCa**										
Locally Advanced (T3a/bT4 N0M0)	N/A	N/A	N/A	N/A	879 (47.3)	137 (26.3)	N/A	N/A	N/A	N/A
Advanced (TxNxM1)	N/A	N/A	N/A	N/A	981 (52.7)	385 (73.8)	N/A	N/A	N/A	N/A
**PCa stage subgroups**										
TxNxM1	N/A	N/A	N/A	N/A	981 (52.7)	385 (73.8)	N/A	N/A	N/A	N/A
TxN1M0	N/A	N/A	N/A	N/A	315 (16.9)	60 (11.5)	N/A	N/A	N/A	N/A
T3aNxMx	N/A	N/A	N/A	N/A	287 (15.4)	25 (4.8)	N/A	N/A	N/A	N/A
T3bNxMx	N/A	N/A	N/A	N/A	153 (8.2)	15 (2.9)	N/A	N/A	N/A	N/A

T4NxMx	N/A	N/A	N/A	N/A	124 (6.7)	37 (7.1)	N/A	N/A	N/A	N/A
T3 (Netherlands)	N/A	N/A	N/A	N/A	N/A	N/A	417 (35.8)	9 (9.6)	N/A	N/A
T4 (Netherlands)	N/A	N/A	N/A	N/A	N/A	N/A	748 (64.2)	85 (90.4)	N/A	N/A
Missing	N/A	N/A	N/A	N/A	0	0	22 (1.9)	3 (3.1)	N/A	N/A
**Total Gleason Score**										
Gleason 5-6	N/A	N/A	989 (10.9)	25 (5.1)	N/A	N/A	52 (4.4)	4 (4.1)	N/A	N/A
Gleason 7	N/A	N/A	3023 (33.2)	72 (14.5)	N/A	N/A	228 (19.2)	14 (14.4)	N/A	N/A
Gleason 8	N/A	N/A	1154 (12.7)	40 (8.1)	N/A	N/A	194 (16.3)	19 (19.6)	N/A	N/A
Gleason 9-10	N/A	N/A	2139 (23.5)	257 (51.9)	N/A	N/A	240 (20.2)	22 (22.7)	N/A	N/A
Missing	N/A	N/A	2815 (30.9)	374 (75.6)	N/A	N/A	473 (39.9)	38 (39.2)	N/A	N/A
**PSA**										

≤10	N/A	N/A	N/A	N/A	N/A	N/A	192 (16.2)	8 (8.3)	N/A	N/A
11-20	N/A	N/A	N/A	N/A	N/A	N/A	186 (15.7)	7 (7.2)	N/A	N/A
21-50	N/A	N/A	N/A	N/A	N/A	N/A	237 (20.0)	12 (12.4)	N/A	N/A
>50	N/A	N/A	N/A	N/A	N/A	N/A	479 (40.4)	51 (52.6)	N/A	N/A
Missing	N/A	N/A	N/A	N/A	N/A	N/A	93 (7.8)	19 (19.6)	N/A	N/A
**Any prior PCa treatment**										
Radical prostatectomy	292 (1.7)	3 (2.5)	229 (3.0)	<5.0	51 (2.7)	6 (1.2)	62 (5.2)	2 (2.1)	1000 (5.1)	35 (3.8)
Radical prostatectomy + Adjuvant/Salvage Radiotherapy	N/A	N/A	N/A	N/A	14 (0.8)	1 (0.2)	N/A	N/A	29 (0.2)	1 (0.11)
Radiotherapy	305 (1.8)	3 (2.5)	4281 (47.0)	145 (29.0)			403 (34.0)	9 (9.3)	269 (1.37)	13 (1.42)
Radiotherapy ≤ 6 months prior to ADT initiation	N/A	N/A	N/A	N/A	N/A	N/A	N/A	N/A	55 (20.5)	4 (30.8)
Radiotherapy > 6 months prior to ADT initiation	N/A	N/A	N/A	N/A	N/A	N/A	N/A	N/A	214	9

									(79.5)	(69.2)
Palliative radiotherapy (1-10 fractions)	N/A	N/A	N/A	N/A	108 (5.8)	67 (12.8)	N/A	N/A	N/A	N/A
Long course external beam radiotherapy +/- brachytherapy	N/A	N/A	N/A	N/A	453 (24.4)	56 (10.7)	N/A	N/A	N/A	N/A
Chemotherapy	N/A	N/A	N/A	N/A	N/A	N/A	14 (1.2)	1 (1.0)	N/A	N/A
AA	4214 (24.9)	7 (5.9)	N/A	N/A	990 (53.2)	42 (8.1)	1037 (87.4)	67 (69.1)	N/A	N/A
Previous AA 0-3 month	N/A	N/A	N/A	N/A	N/A	N/A	N/A	N/A	1669 (87.8)	39 (92.9)
Previous AA 3-6 month	N/A	N/A	N/A	N/A	N/A	N/A	N/A	N/A	145 (7.6)	1 (2.4)
Previous AA 6-9 month	N/A	N/A	N/A	N/A	N/A	N/A	N/A	N/A	71 (3.7)	1 (2.4)
Previous AA 9-12 month	N/A	N/A	N/A	N/A	N/A	N/A	N/A	N/A	16 (0.8)	1 (2.4)
Brachytherapy	N/A	N/A	N/A	N/A	N/A	N/A	N/A	N/A	N/A	N/A
EBRT	N/A	N/A	N/A	N/A	N/A	N/A	N/A	N/A	N/A	N/A
Other/none	12144	105			244	350	N/A	N/A	N/A	N/A

	(71.6)	(89.0)			(13.1)	(67.1)				
**ADT specifics (with GnRH agonists)**										
Anti-androgens – flare protection	3764 (22.2)	4 (3.4)	N/A	N/A	506 (27.2)	13 (2.5)	402 (33.9)	20 (20.6)	8527 (43.4)	41 (4.5)
Anti-androgens – combined androgen blockade	276 (1.6)	1 (0.9)	N/A	N/A	953 (51.2)	44 (8.4)	635 (53.5)	47 (48.5)	4199 (21.4)	53 (5.8)
No anti-androgens	12741 (75.1)	111 (94.1)	N/A	N/A	401 (21.6)	465 (89.1)	150 (12.6)	30 (30.9)	6805 (34.7)	795 (87.2)
Unknown*	174 (1.03)	2 (1.7)	N/A	N/A	0	0			11 (0.56)	23 (2.5)
**CVD risk factors 12 months prior to ADT initiation**										
Hypertension	5729 (33.8)	42 (35.6)	5375 (59.0)	319 (64.4)	1124 (60.4)	280 (53.6)	481 (40.5)	47 (48.5)	10251 (52.2)	457 (50.1)
Dyslipidaemia	4547 (26.8)	36 (30.5)	4224 (46.3)	268 (54.1)	714 (38.4)	197 (37.7)	446 (37.6)	32 (33.0)	8852 (45.1)	369 (40.5)
Diabetes Mellitus	1173 (6.9)	11 (9.3)	912 (10.0)	64 (12.9)	277 (14.9)	97 (18.6)	163 (13.7)	10 (10.3)	3343 (17.0)	152 (16.7)

**Number of previous CVD events, 12 months prior to ADT initiation**										
0	16540 (97.6)	113 (95.8)	8725 (95.7)	451 (91.1)	1551 (83.4)	437 (83.7)	1128 (95.0)	91 (93.8)	18541 (94.4)	838 (91.9)
1	98 (0.6)	2 (1.7)	158 (1.7)	16 (3.2)	155 (8.3)	50 (9.6)	18 (1.5)	1 (1.0)	944 (4.8)	66 (7.2)
2	119 (0.7)	0	111 (1.2)	10 (2.0)	92 (5.0)	25 (4.8)	5 (0.4)	2 (2.1)	130 (0.7)	6 (0.7)
3+	198 (1.2)	3 (2.5)	120 (1.3)	18 (3.6)	62 (3.3)	10 (1.9)	36 (3.0)	3 (3.1)	26 (0.1)	2 (0.2)
**Time of last previous CVD, 12 months prior to ADT initiation**										
No CVD	16540 (97.6)	113 (95.8)	N/A	N/A	1551 (83.4)	437 (83.7)	1128 (95.0)	91 (93.8)	18541 (94.4)	838 (91.9)
0-3m	141 (0.8)	3 (2.5)	N/A	N/A	254 (13.7)	71 (13.6)	16 (1.4)	3 (3.1)	365 (1.9)	32 (3.5)
4-6m	98 (0.6)	0	N/A	N/A	29 (1.6)	8 (1.5)	15 (1.3)	0	274 (1.4)	14 (1.5)
7-12m	176 (1.0)	2 (1.7)	N/A	N/A	26 (1.4)	6 (1.2)	28 (2.4)	3 (3.1)	461 (2.4)	28 (3.1)

**BMI / obesity**										
Normal weight (18.5-24)	76	2	N/A	N/A	N/A	N/A	N/A	N/A	N/A	N/A
	(0.5)	(1.7)								
Underweight (<18.5)	2	0	N/A	N/A	N/A	N/A	N/A	N/A	N/A	N/A
	(0.01)									
Overweight (25-30)	165	2	N/A	N/A	N/A	N/A	N/A	N/A	N/A	N/A
	(1.0)	(1.7)								
Obese (>30)	103	0	N/A	N/A	N/A	N/A	N/A	N/A	N/A	N/A
	(0.6)									
Missing	16609	114	N/A	N/A	N/A	N/A	N/A	N/A	N/A	N/A
	(98.0)	(96.6)								
**Socio-economic Status**										
Lowest or least deprived (Townsend 1)	3402	18	1380	105	N/A	N/A	N/A	N/A	N/A	N/A
	(20.1)	(15.3)	(15.1)	(21.2)						
Low (Townsend 2)	2638	32	1708	108	N/A	N/A	N/A	N/A	N/A	N/A
	(15.6)	(27.1)	(18.7)	(21.8)						
Middle (Townsend 3)	2223	16	1939	113	N/A	N/A	N/A	N/A	N/A	N/A
	(13.1)	(13.6)	(21.3)	(22.8)						
High (Townsend 4)	1700	18	2132	95	N/A	N/A	N/A	N/A	N/A	N/A
	(10.0)	(15.3)	(23.4)	(19.2)						

Highest or most deprived (Townsend 5)	975 (5.8)	10 (8.5)	1951 (21.4)	73 (14.7)	N/A	N/A	N/A	N/A	N/A	N/A
French "poor income"	N/A	N/A	N/A	N/A	N/A	N/A	N/A	N/A	550 (2.8)	22 (2.4)
Missing	6017 (35.5)	24 (20.3)	N/A	N/A	N/A	N/A	N/A	N/A	N/A	N/A
**Civil Status**										
Single	114 (0.7)	2 (1.7)	N/A	N/A	N/A	N/A	N/A	N/A	N/A	N/A
Married	556 (3.3)	5 (4.2)	N/A	N/A	N/A	N/A	N/A	N/A	N/A	N/A
Missing	16285 (96.1)	111 (94.1)	N/A	N/A	N/A	N/A	N/A	N/A	N/A	N/A
**Smoking Status**										
Current Smokers	3729 (22.0)	30 (25.4)	N/A	N/A	N/A	N/A	N/A	N/A	N/A	N/A
Non-smokers	78 (0.5)	0	N/A	N/A	N/A	N/A	N/A	N/A	N/A	N/A
Past Smokers	195 (1.2)	0	N/A	N/A	N/A	N/A	N/A	N/A	N/A	N/A

Missing	12953	88	N/A	N/A	N/A	N/A	N/A	N/A	N/A	N/A
	(76.4)	(74.6)								
**Ethnicity**										
Caucasian	7392	57	N/A	N/A	N/A	N/A	N/A	N/A	N/A	N/A
	(43.6)	(48.3)								
Black	360	0	N/A	N/A	N/A	N/A	N/A	N/A	N/A	N/A
	(2.1)									
Asian	226	1	N/A	N/A	N/A	N/A	N/A	N/A	N/A	N/A
	(1.3)	(0.9)								
Other	152	1	N/A	N/A	N/A	N/A	N/A	N/A	N/A	N/A
	(0.9)	(0.9)								
Missing	8825	59	N/A	N/A	N/A	N/A	N/A	N/A	N/A	N/A
	(52.1)	(50.0)								

*Unknown: Received anti-androgens before GnRH initiation however cannot make a distinction between flare or combined androgen blockade; N/A: Not available

Supplement Table 2: Hazard ratios from sensitivity analysis excluding UK using random-effects meta-analytical model including different stratifications any CVD, ischaemic heart disease, acute myocardial infarction, arrhythmia, heart failure and stroke for Scotland, Belgium, the Netherlands and France.

Outcome	HR (95% CI)	HR for PCa men with *HCVDi (95% CI)	HR for PCa men without HCVDi (95% CI)	HR for PCa men < 75 years (95% CI)	HR for PCa men ≥ 75 years (95% CI)
Any CVD	1.21 (0.93 – 1.58)	1.29 (1.01 – 1.64)	1.05 (0.76 – 1.44)	1.28 (0.97 – 1.70)	1.15 (0.89 – 1.47)
Ischaemic Heart Disease	1.23 (0.93 – 1.63)	1.22[*1] (0.87 – 1.73)	1.83 (0.97 – 3.45)	1.17 (0.81 – 1.69)	1.32[*1] (0.93 – 1.87)
Acute Myocardial Infarction	1.62[*1] (1.11 – 2.35)	1.63[*1] (1.09 – 2.43)	2.05[*2] (0.75 – 5.62)	2.16[*1] (1.27 – 3.67)	1.31[*1] (0.77 – 2.22)
Arrhythmia	1.47 (1.10 – 1.96)	1.70 (1.27 – 2.28)	3.11[*3] (0.28 – 35.25)	1.55 (0.98 – 2.45)	1.39[*1] (0.96 – 2.02)
Heart Failure	1.38 (1.00 – 1.91)	1.36[*1] (0.96 – 1.93)	2.45[*4] (0.85 – 7.05)	1.71[*1] (0.91 – 3.21)	1.27 (0.86 – 1.88)
Stroke	0.90 (0.61 – 1.33)	0.86[*1] (0.54 – 1.36)	1.31[*1] (0.60 – 2.86)	0.79[*1] (0.30 – 2.06)	0.96 (0.61 – 1.52)

GnRH agonists is the reference group in all analyses; * history of CVD indicator was defined as a prescription or dispensation of medication for any of the following 12 months prior to entering the cohort: any CVD event, hypertension, dyslipidaemia or diabetes; *1. Netherlands was excluded due to low number of events for country-specific analysis; *2. Belgium and the Netherlands were excluded due to low number of events for country-specific analysis; *3. Scotland, the Netherlands and France were excluded due to low number of events for country-specific analysis; *4. Scotland and the Netherlands were excluded due to low number of events for country-specific analysis.

Supplement Table 3: Hazard ratios from sensitivity analysis for 3 months using random-effects meta-analytical model including different stratifications any CVD, ischaemic heart disease, acute myocardial infarction, arrhythmia, heart failure and stroke for the United Kingdom, Scotland, Belgium, the Netherlands and France.

Outcome	HR (95% CI)	HR for PCa men with *HCVDi (95% CI)	HR for PCa men without HCVDi (95% CI)	HR for PCa men < 75 years (95% CI)	HR for PCa men ≥ 75 years (95% CI)
**Any CVD**	1.10 (0.86 – 1.41)	1.19 (0.95 – 1.49)	0.84 (0.54 – 1.31)	1.16 (0.89 – 1.52)	1.07 (0.86 – 1.33)
**Ischaemic Heart Disease**	1.23[*1] (0.91 – 1.65)	1.26[*2] (0.92 – 1.73)	1.64[*3] (0.63 – 4.29)	1.22[*4] (0.68 – 2.18)	0.84[*3] (0.46 – 1.52)
**Acute Myocardial Infarction**	1.64[*2] (1.05 – 2.55)	1.71[*2] (1.07 – 2.73)	1.40[*5] (0.19 – 10.59)	2.71[*4] (1.19 – 6.16)	1.36[*4] (0.47 – 3.98)
**Arrhythmia**	1.17 (0.70 – 1.94)	1.34 (0.84 – 2.13)	3.04[*5] (0.27 – 34.53)	1.55 (0.82 – 2.95)	1.02[*1] (0.61 – 1.69)
**Heart Failure**	1.38 (0.94 – 2.03)	1.37[*1] (0.94 – 2.09)	2.14[*4] (0.60 – 7.58)	1.92[*4] (0.92 – 4.03)	1.04[*6] (0.57 – 1.90)
**Stroke**	1.00[*7] (0.62 – 1.60)	0.92[*2] (0.48 – 1.78)	2.28[*4] (0.59 – 8.84)	1.16[*4] (0.13 – 10.41)	1.08[*8] (0.57 – 2.05)

GnRH agonists is the reference group in all analyses; * history of CVD indicator was defined as a prescription or dispensation of medication for any of the following 12 months prior to entering the cohort: any CVD event, hypertension, dyslipidaemia or diabetes; *1. The Netherlands was excluded due to low number of events for country-specific analysis; *2. UK and the Netherlands were excluded due to low number of events for country-specific analysis; *3. Scotland and the Netherlands were excluded due to low number of events for country-specific analysis; *4. UK, Scotland and the Netherlands were excluded due to low number of events for country-specific analysis; *5. UK, Scotland, Belgium and the Netherlands were excluded due to low number of events for country-specific analysis; *6. Scotland was excluded due to low number of events for country-specific analysis; *7. UK was excluded due to low number of events for country-specific analysis; *8. UK and Scotland were excluded due to low number of events for country-specific analysis.

Supplement Table 4: Data extraction in the United Kingdom, Scotland, Belgium, the Netherlands and France.

Study Variables	Data Extraction in the United Kingdom, Scotland, Belgium, the Netherlands and France
**Age**	Age was considered as a timescale in all analytical models on the date of study entry (i.e. GnRH agonists or antagonists' initiation). Age was defined as: ≤ 65, 66-74, 75-84, ≥ 85 and missing. Multiple imputation was used to impute missing age.
**Follow-up Time**	Follow-up for the study started on the date of treatment initiation and ended when they reached any of the censoring criteria. The median, lower and upper quartiles for follow-up time were calculated for all five countries.
**Year of PCa Diagnosis**	Year of PCa diagnosis was defined as: ≤2010, 2011, 2012, 2013, 2014, 2015, 2016 and missing.
**Stage of PCa**	PCa stage at diagnosis was defined as: locally advanced (T3a/bT4 N0M0) and metastatic (TxNxM1). In Belgium, the categories were: TxNxM1, TxN1M0, T3aNxMx, T3bNxMx and T4NxMx.
**Total Gleason Score**	Total Gleason score was defined as: Gleason 5-6, 7, 8, 9-10 and missing.
**Prostate Specific Antigen (PSA)**	PSA defined as: ≤10, 11-20, 21-50 and >50 ng/ml.
**Any Prior PCa Treatment**	The main categories were defined as: radical prostatectomy, radiotherapy and anti-androgens. In Belgium, radical prostatectomy and adjuvant or salvage radiotherapy was an additional group. Chemotherapy was included in the Netherlands included brachytherapy and external beam therapy.
**Type of ADT**	Type of ADT was defined so that GnRH agonists or antagonists as to distinguish whether GnRH was given as a primary, adjuvant, neo-adjuvant treatment or other (Belgium only).
**ADT Specifics**	ADT specifics was defined as: men who had received anti-androgens as flare protection (anti-androgens for ≤ 30 days) or combined androgen blockade (anti-androgens for > 30 days).

**History of CVD Indicator**	History of CVD indicator was defined as: any of the following 12 months prior to entering the cohort: any CVD event (ICD-10 codes: I20-I99, G45), hypertension (ICD-10 and ATC codes), dyslipidaemia (ATC codes or drugcodes) or diabetes (ATC codes or drugcodes). History of CVD indicator was further sub-categorised to specifically indicate history of hypertension, dyslipidemia or diabetes 12 months prior to study entry.
**Number of Previous CVD Events**	The number of CVD events prior to entering the cohort were coded as 0, 1, 2 or ≥ 3 CVD events.
**Other Socio-Demographics**	Body Mass Index (BMI), socio-economic status (SES), civil status, smoking status and ethnicity were extracted in the UK using the readcodes. BMI was defined as: underweight at ≤ 18.5 kg/m^2, normal at 18.6-24 kg/m^2, overweight at 25-30 kg/m^2 and obese at ≥ 30 kg/m^2.<br><br>Townsend scores define SES in the study population. Townsend scores incorporated four different variables: unemployment, non-car ownership, non-home ownership and household overcrowding. The Townsend scores were given as quintiles (i.e. five groups of equal size ranging from 1 (least deprived) to 5 (most deprived).<br><br>In THIN, civil status was defined as: single, married and unknown.<br><br>Smoking status was defined as: current smokers, non-smokers and past smokers.<br><br>Ethnicity was defined as men with an origin of: Caucasian, Black, Asian and other (readcodes excluding these three categories).
